# An urgent need for longitudinal microbiome profiling coupled with machine learning interventions

**DOI:** 10.3389/fmicb.2024.1487841

**Published:** 2024-11-11

**Authors:** Priyankar Dey, Sandeep Choubey

**Affiliations:** ^1^Department of Biotechnology, Thapar Institute of Engineering and Technology, Patiala, India; ^2^The Institute of Mathematical Sciences, Chennai, India; ^3^Homi Bhabha National Institute, Mumbai, India

**Keywords:** liver, microbiome, gut-liver axis, machine learning, artificial intelligence, microbiota, gut, intestine

## 1 Introduction: lack of universal gut microbial signatures associated with metabolic liver disease

Our understanding of the role of gut microbes in human health and disease has come a long way since John M. Whipps et al. first defined the term “microbiome.” Since the early 2000s, with the gradual lowering of the cost of commercial DNA sequencing, health science has been flooded with 16S rRNA data. Unfortunately, despite a plethora of pre-clinical and clinical publications on the gut–liver axis, the majority of our understanding of the microbiota–liver reciprocal interaction remains limited to correlation analysis. The most exciting part of such metagenomic studies is the sheer amount of “big data” generated, which is rather easy to correlate with physiological variables; the bigger the metagenomic data and number of independent variables, the more the chances to find “significant” associations. Notably, our limited understanding of the gut–liver axis is derived from the microbiome, rather than the microbiota. Therefore, the conclusions obtained through microbiome-related correlation studies often do not reflect causation and are not representative of a universal phenomenon, and there have been almost no true microbial markers of dysbiosis linked to chronic liver disease.

## 2 Discussion

### 2.1 The boundary between the known and the unknown

Translational potentials of pre-clinical microbiota–liver associations in clinical disease prediction and treatment have not been very successful despite enormous numbers of interventional and observational studies. In fact, the relevance of utilizing rodents' gut microbial signatures in understanding human diseases has been criticized due to the massive difference in the gut microbes between both species, attributed to the gastrointestinal biogeography and genetic makeup (Nguyen et al., 2015). Especially in the high-fat diet model of metabolic disease, there is a lack of consistency between good and bad microbes. In fact, diseases have also been associated with the strains belonging to probiotics (e.g., *Lactobacillus*) and commensals (e.g., *Akkermansia* and *Faecalibacterium*) (Dey and Ray Chaudhuri, 2023). Furthermore, confounding results are obtained from chemical-induced metabolic disease models that are physiologically mostly irrelevant (Dey, 2020) and data from germ-free mice that possess innate defects in various physiological processes (Jans and Vereecke, 2024). The clinical progression of metabolic liver disease is distinct from pre-clinical models of chronic liver disease in terms of timeline, cellular phenotype, pathological complexity, the difference in immune responses, and metabolic machinery (Liu et al., 2013). Furthermore, the innate and general differences between human and animal disease models, such as biological differences (e.g., genetic makeup, organ anatomy, and liver functional capacity), extent of disease complexity (e.g., model specificity and co-influence of comorbidities), ability to perform controlled experiments, and predictive validity, make it challenging to conclude on clinical gut microbial phenotypes based on pre-clinical data. Although there have been emerging reports of *in vitro* models of the human distal intestine (Qi et al., 2023), these models are physiologically irrelevant given their host-independent nature.

Due to the advent of culturomics techniques and controlled clinical studies, pre-clinical gut microbial patterns that were initially considered associated with disease conditions, such as Firmicutes-to-Bacteroides ratio, enrichment of energy-harvesting species, and specific metabolic functions, seem to be falling apart. For instance, γ-proteobacteria, due to the presence of lipopolysaccharide (LPS), were previously thought to simply cause hepatic inflammation. However, studies have identified that the LPS–TLR4–inflammation axis cannot be generalized due to huge differences in LPS structure dictating the extent of immune response (Picarello, 2022) and that the majority of the luminal LPS-supplying Bacteroides rather display immunosuppressive characteristics (d'Hennezel et al., 2017). Today, it is strongly recognized that the good, bad, and ugly nature of the gut microbiota is condition-specific. Factors such as the availability of preferred nutrients, pathoadaptive mutations, and potential to evade the mucosal immune response have been recognized as critical factors that define a specific microbial species as commensal or pathobiont (Dey and Ray Chaudhuri, 2023; Dey, 2024). The bottom line is that there is no proper clinical definition of dysbiosis and eubiosis in terms of specific microbial features. However, the only aspect almost universally accepted is that loss of gut microbial diversity is associated with chronic liver disease, and when we talk about the diversity, it is the community effects not disease causation by a single species.

### 2.2 A critical lack of liver-specific longitudinal studies

A recent systematic review and meta-analysis of 54 clinical studies have indicated substantial inter-study heterogeneity in gut microbial taxonomic identification, in which the enrichment of inflammation-inducing genera was more closely associated with non-alcoholic fatty liver disease, but no genera were identified to provide long-term disease risk-predictive value (Su et al., 2024). To date, there have been more than 100 cross-sectional studies to identify the predominant gut microbes associated with metabolic liver disease, yet no absolute core microbiome related to progressive liver disease has been identified. Although longitudinal studies are considered superior to cross-sectional studies and that informed understanding of disease pathogenesis can only be obtained through the former, there have been only a few longitudinal studies undertaken to link the gut microbiome with liver health. One study from Kyoto (Japan) evaluated the gut microbial alterations from pre-transplantation to 2 months post-surgery in 38 liver transplant patients (Kato et al., 2017). Data show an initial decline and later increase of microbial diversity, along with the overall increase of Bacteroides, Enterobacteriaceae, Streptococcaceae, and Bifidobacteriaceae, while a depletion in the abundance of Lactobacillaceae, Enterococcaceae, Clostridiaceae, Peptostreptococcaceae, and Ruminococcaceae was noted. The authors acknowledged that variations in antibiotic regimes, food, synbiotics, and patient heterogeneity (e.g., various donors and underlying diseases) likely influence the study's findings. Confounding variables and data on relative microbial abundance were among the limitations. In line, a relatively recent study from the National Institutes of Health, USA, investigated the gut–liver axis in hepatitis C patients, taking into account varied degrees of fibrosis severity (Ali et al., 2023). The investigation was performed 6 months after HCV was undetectable (*n* = 23) and before (*n* = 29) attaining a durable virologic response. Data suggest that increased hepatic fibrosis was correlated with *Anaerostipes hadrus*, while *Bacteroides vulgatus* with portal inflammation in HCV. A prolonged virologic response suggests that *Methanobrevibacter smithii* may have a beneficial effect on indicators of the severity of liver disease. Although this longitudinal investigation made it possible to compare HCV-infected individuals with a supposedly improved viral clearance state, they were unable to report gut microbial patterns in healthy controls due to the unavailability of samples. Another recent study from Stanford examines the gut microbial composition at various body sites (including gut) and correlated with host multi-omics, immunological, and clinical indicators (including hepatic) (*n* = 86) over 6 years to comprehend the dynamic interaction between the human microbiomes and host throughout health and illness (Zhou et al., 2024). Despite identifying microbial compositional and diversity patterns associated with host physiological parameters and metabolites, no temporal associations were derived between the gut microbiota and the measured liver-specific parameters (e.g., transaminases). Emerging studies claim the causative effects of oral microbiota in the pathogenesis of chronic metabolic disease, including liver disease (Gupta and Dey, 2023), and there has been no longitudinal study undertaken in this line to date. Thus, understanding the true nature of gut microbial dynamics under the course of hepatic disease pathogenesis and remission remains largely unknown.

### 2.3 Machine learning in pattern recognition along the gut–liver axis

Beyond the availability of longitudinal gut microbial data, a fundamental difficulty in analyzing large-scale microbiome big-data lies in their high dimensionality (Advani and Ganguli, 2016). In classically designed experiments, a small number of carefully selected variables (V) are measured to test a specific hypothesis, with a large number of measurements (M) for each variable. Thus, the measurement density is very large (M/V → ∞). Such datasets are referred to as low-dimensional, and much of classical statistics operates within this framework. In contrast to this classical scenario, the recent technological capacity for high-throughput sequencing has led to a different statistical regime. It is commonplace to simultaneously measure many variables (V), such as the abundance of hundreds of taxa at the individual level. However, due to constraints on time or resources, often it is possible only to make a limited number of simultaneous measurements. Thus, while both M and V are large, the measurement density (M/V) is much smaller than in conventional experiments. Such datasets are referred to as high dimensional, that is, they consist of a small number of points in a high-dimensional space. Microbiome datasets consist of the composition of thousands of microbial taxa in an individual gut. Hence, understanding the role of gut microbiome in liver health requires us to use machine learning (ML) approaches to dissect such a high-dimensional dataset. Utilizing these approaches enhances our understanding of the complex host–microbe reciprocal, helping in tracking disease progression over time or monitoring treatment responses, which is valuable for personalized medicine.

In recent years, ML approaches have been widely used to shed light on how gut microbiome impacts various liver diseases. These studies have identified microbiome biomarkers associated with metabolic liver diseases (Ruuskanen et al., 2021; Zhang et al., 2021; Liu et al., 2022; Park et al., 2024). However, not only that majority of these studies are mostly cross-sectional in nature but also these ML approaches suffer from a number of limitations. First, it is extremely difficult to detect patterns in microbiome datasets that can provide useful biological insight with translational value. High-dimensional data analysis techniques such as PCA, ICA, or t-SNE are useful for reducing dimensionality and detecting patterns. However, since the resulting axes represent linear combinations of a large number of features (e.g., taxa abundance), interpreting the analysis or making experimental predictions is often difficult (Donoho and Tanner, 2009; Furchtgott et al., 2017). Identifying patterns becomes even more challenging as the proportion of relevant features decreases at higher taxonomic levels (Donoho and Tanner, 2009). In fact, decades of research related to the gut microbiome have shown that specific combinations of a few microbes can be associated with a disease. This indicates that the composition of a small subset of microbes may be most relevant for making accurate computational inferences. Therefore, there is a need to develop novel methods to detect sparse patterns in high-dimensional datasets.

The second challenge is to extract meaningful models from high-dimensional datasets that can predict interventional outcomes and guide translational implications of research findings. Building complex models describing microbial networks involves hundreds of parameters. Unfortunately, in most cases, the parameters remain completely unknown. As Von Neumann once said: “*with four parameters I can fit an elephant, and with five I can make him wiggle his trunk*.” The challenges involving microbial networks are clearly exacerbated since even the simplest of models would require hundreds of parameters. Hence, the available data are always limited and can never constrain the space of models completely. Given the underconstrained nature of the problem, there exist an infinite number of combinations of the parameters that can successfully replicate the data. Hence, making predictions based on such underconstrained gut microbial networks has proven to be challenging. Moreover, most of the studies trained their models on specific datasets, such as Western or Chinese datasets, that may not generalize to different populations, limiting their overall applicability. Often these studies used a small patient population size. Some of these studies suffered from the absence of population characteristics such as the lack of lifestyle information (e.g., diet, socioeconomic status, tobacco, and alcoholism). Furthermore, in some cases, the lack of patient clinical, metagenomic, and metabolomic profiles hampered garnering a comprehensive understanding of the various causal aspects of the disease.

## 3 Conclusion

The complexity of the gut microbial dynamics and its function in metabolic liver disease remains largely unknown. The progressive character and course of liver disorders are not well captured by pre-clinical and cross-sectional investigations. We suggest using cutting-edge ML methods in conjunction with longitudinal research to address these issues. Hence, it is imperative to first develop methods to detect sparse relevant features that can then be used to find patterns in the data and train predictive models. By addressing these limitations and building new computational approaches, we can fully harness the potential of ML approaches to deepen our understanding of the gut microbiome's role in liver disease and other areas. By doing this, we can pinpoint certain gut microbial patterns associated with the advancement of liver disease, resulting in the development of more potent preventative and therapeutic approaches. For gut–liver axis research to reach its full potential, machine learning must be included.
